# Bi-rooted Primary Maxillary Canines: A Case Report

**DOI:** 10.5681/joddd.2010.026

**Published:** 2010-09-16

**Authors:** Maryam Talebi, Iman Parisay, Fatemeh Khorakian, Mohammad Bagherian

**Affiliations:** ^1^ Associate Professor, Faculty of Dentistry, Department of Pediatric Dentistry, Mashhad University of Medical Sciences, Mashhad, Iran; ^2^ Assistant Professor, Faculty of Dentistry, Department of Pediatric Dentistry, Shahid Sadooghi University of Medical Sciences, Yazd, Iran; ^3^ Post-graduate Student, Faculty of Dentistry, Department of Pediatric Dentistry, Mashhad University of Medical Sciences, Mashhad, Iran; ^4^ Dentist, Private Practice, Mashhad, Iran

**Keywords:** Bi-rooted canines, morphology, primary teeth

## Abstract

This report presents a rare case of bi-rooted primary maxillary canines. A 6-year-old girl presented at the Department of Pediatric Dentistry, Faculty of Dentistry, Mashhad University of Medical Sciences, Iran, with the chief complaint of pain in the lower right quadrant of her dentition. Radiographic examination revealed bifurcation of primary maxillary canine roots. This report discusses the possible etiology of bi-rooted canines, implications for the developing dentition, and treatment options for these teeth.

## Introduction

Fewer abnormalities of size and morphology occur in the primary dentition compared to permanent teeth.^1^ The primary maxillary canine normally possesses a single conical root.^1^ Bi-rooted primary canines are very rare; only a few cases have been reported in Japanese, African-American, Caucasian and Pueblo Indian children.^2 - 6^The dental literature contains many articles reporting anomalies of the permanent dentition. There are especially fewer primary radicular anomalies compared to permanent radicular anomalies.^7^

This report describes a case of bi-rooted primary maxillary canines in an Iranian girl, in which the bifurcations extended mesially and distally to increase the awareness of morphological aberrations of this situation.

## Case Report

**Figure 1:**
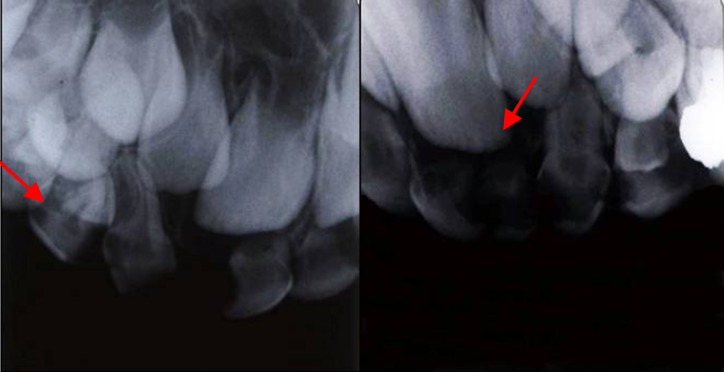
Periapical radiographic view of the double-rooted maxillary primary canines.

**Figure 2:**
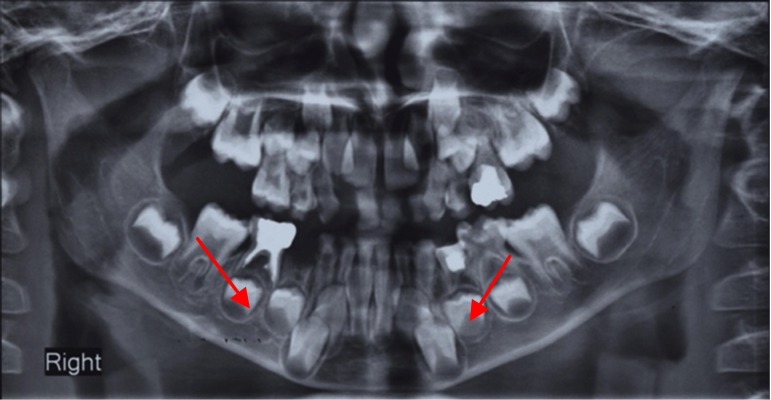
Panoramic view of the patient.

A 6-year-old girl was brought to the Department of Pediatric Dentistry, Faculty of Dentistry, Mashhad University of Medical Sciences, Mashhad, Iran, with the chief complaint of lower right molar pain. The patient’s medical history did not reveal any systemic abnormality or congenital disease. Intra-oral examination revealed a fully erupted, early mixed dentition with multiple deep carious lesions in several primary teeth. Oral hygiene instruction and dietary recommendations were first provided in an attempt to counteract the patient’s poor oral hygiene and high incidence of caries. Clinical and radiographic examinations indicated that the primary right mandibular first molar and left maxillary first molar needed to be extracted, and that root canal therapy and restorations were necessary for many teeth (including the maxillary canines). Periapical and panoramic radiographs did not clearly show that maxillary canines were double-rooted, with clinical examination showing normal canine shape and size (Figures 1 and 2). However, radiographic images revealed mesiodistally expanded pulp chambers and bifurcated roots (Figures 1 & 2). The bifurcation was located at the lower third of the roots, with mesiodistal apical divergence. This divergence was particularly pronounced in the maxillary left canine.

**Figure 3:**
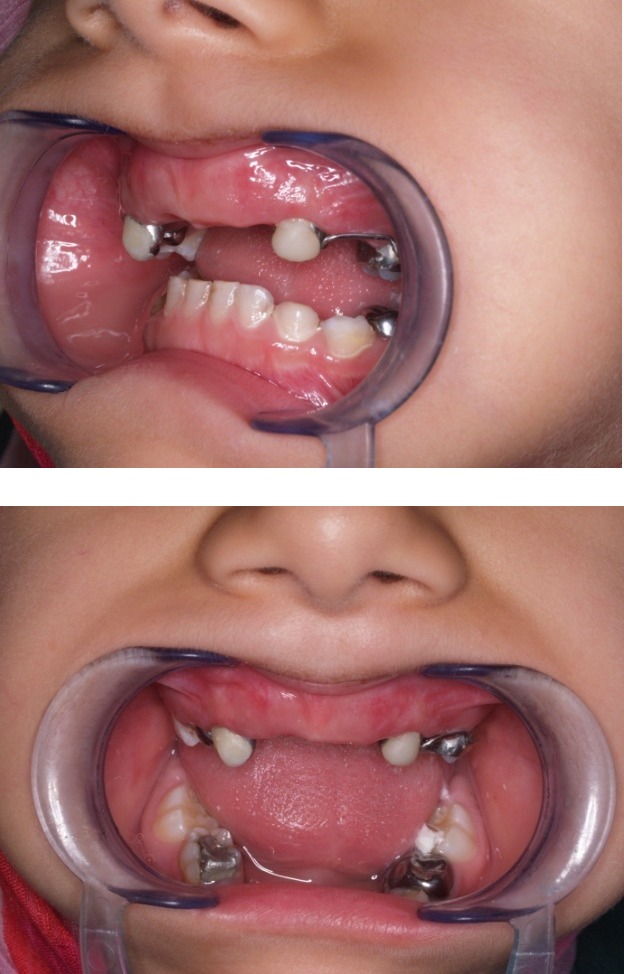
Intra-oral photographs of the patient.

**Figure 4:**
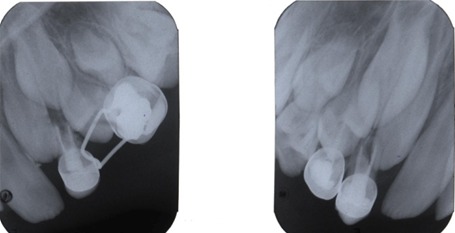
Periapical radiographs after treatment of canines.

Parental consent was obtained to proceed with the treatment approach. Both maxillary canines exhibited deep inter-proximal carious lesions with pulp chamber involvement. These lesions were treated with pulpectomy and stainless steel crowns (Figures 3 & 4).

The mandibular canines were found to be single-rooted.

## Discussion

Previously reported cases of bi-rooted primary canines are summarized in Table 1. A literature review revealed a high tendency for this anomaly to occur bilaterally in males in the maxilla (Table 1). The case reported here affected an Iranian female, and the anomaly was bi-lateral in the maxilla.

The etiology of supernumerary roots in the human dentition is poorly understood. Kelly (1978) suggested that bifurcated roots may be related to the medial growth of Hertwig's epithelial root sheath (HERS).^8^ Formed by the enamel organ, HERS moulds the shape of roots and initiates dentin formation. The sheath develops differently in single- and multi-rooted teeth.^4^ In the early stages of root formation, a defect in the dental lamina may contribute to the development of bi-rooted canines. Other researchers have suggested that fusion or germination may be related to the clinical presentation of supernumerary roots.^7 - 11^ Abnormalities in the morpho-differentiation of canines may also be etiological factors. Such abnormalities may be genetically determined or be associated with environmentally induced cellular changes.^2^For normal exfoliation to occur, the permanent successor must resorb the roots evenly. The anomaly of the permanent canine or eruption of the permanent canine may not lead to normal exfoliation of the primary canine. This unusual root anomaly can lead to endodontic and extraction complications, as well as problems in permanent tooth eruption. It should be kept in mind during endodontic therapy that the number of root canals may exceed the number of roots. Observation of bi-rooted primary canines during growth and development may prevent subsequent problems.

## Conclusion

While bi-rooted primary canines cannot be detected by routine intra-oral examination, they are radiographically apparent. This unusual root anatomy can lead to endodontic and extraction complications. Observation of bi-rooted primary canines during growth and development may prevent subsequent problems.
